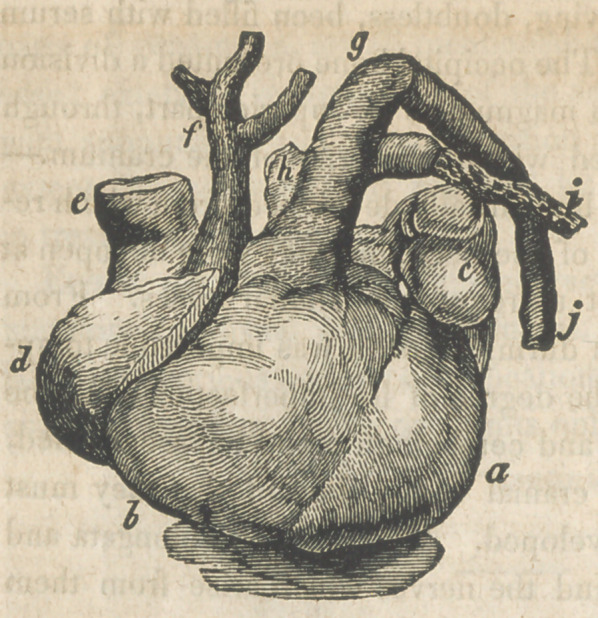# Account of an Anencephalous Fœtus, with an Unusual Malformation of the Heart

**Published:** 1844-05

**Authors:** Daniel Brainard


*Account of an Anencephalous Fœtus, with an unusual malformation of the Heart.* By Daniel Brainard, M. D.

*Dec.* 1843.—I was allowed, by the politeness of Dr. Chas. V. Dyer, of this city, to make an examination of a Fœtus affected with the above named monstrosity, which he had met with in his practice. It was of the female sex, born at the full term of gestation, of healthy parents, who were middle aged, and had six children, all well formed and some of them of uncommon beauty. The limbs and trunk of the one in question were perfectly formed, of medium size, and the subcutaneous cellular and adipose tissues abundant.

The neck was very short, so that the head had the appearance of being attached immediately to the trunk; the shoulders projecting and in contact with the ears; the cranium small, and greatly flattened superiorly, and from its posterior part extended a sac 8 inches in length, and 9 in circumference at its largest part, which was near its extremity. This sac, at its base, was covered by skin like that of the hairy scalp of a fœtus, but near its extremity it was thin, smooth, and destitute of hair. The face was large, the chin projecting, the orbits directed upward, the eyes large and prominent, the frontal and parietal bones receding directly backward. This prominence of the chin, and want of development of the cranium, caused the face to look very much upward.

On laying open the sac, which had been ruptured during labor at its point of connexion with the neck, its walls were found to be composed, externally, of the coverings of the cranium, lined by the membranes of the brain; a part of its cavity was occupied by the brain, the remainder having, doubtless, been filled with serum previously to its rupture. The occipital bone presented a division extending from the foramen magnum to its superior part, through which the sac communicated with the interior of the cranium.—The cervical vertebræ were but partially developed, from which resulted the apparent absence of the neck, but they were not open at the posterior part, as is not unfrequent in similar cases. From the injury done to the brain during labor, it was impossible to appreciate with exactitude, the degree of its imperfection; but the existence of the cerebrum and cerebellum could be ascertained, while the small size of the cranial cavity showed that they must have been but partially developed. The medulla oblongata and spinal cord were perfect, and the nerves which arise from them were distinctly seen.

The above described monstrosity constitutes a variety of those called "*anencephalous*," (from *a. priv.* and *enkephalos*, the brain,) which "consists less in the total absence of the brain and the bones of the cranium, than in the partial want, or imperfect development of these parts." *(Breschet.)* These were for a long time confounded with the "*Acephalous*" (without a head,) from which they are now separated, but to which many of them bear a strong analogy; for if a certain number be taken, they form a progressive series of vicious conformations, from those in which there is a limited opening of the cranial bones with partial deficiency of the brain, to those in which these parts, with the spinal cord, are entirely wanting. The occurrence of this malformation is not, by any means, of very rare occurrence, but the following anomalous arrangement of the great vessels arising from the heart and communication of the ventricles of that organ, *coinciding with it*, has, we believe, rarely been noticed. The size, form, and situation of the organ were normal, but on an attentive examination, it was observed that the pulmonary artery greatly exceeded the aorta in size; that the latter, after taking its origin, as usual, from the left ventricle, formed an ascending portion and divided into two terminal branches, one the brachio cephalic trunk for the right side of the head and the right arm, and the other for the same parts of the left side, while the descending aorta was formed by the continuous trunk of the pulmonary artery. The figure will exhibit this arrangement distinctly.

*a* is the left ventricle; *b* the right ventricle; *c* the left auricle; *d* the right auricle, *e* the descending cava; *f* the ascending aorta dividing into its terminal branches; *g* the pulmonary artery; *h* the branch for the right lung; *i* the branch for the left, arising higher up than the other, with a piece of the lung attached to it; *j* the descending aorta, formed by the continuation of the pulmonary artery. The veins terminating at the heart were natural, except that there was but one pulmonary vein on the left side. No communication by the ductus arteriosus, existed between the aorta and pulmonary artery.

On laying open the cavities of the heart, the auricles were seen to communicate by the foramen ovale, which was of unusual size. The septum of the ventricles was also perforated at its superior part, by an opening a line in breadth by 2½ lines in length. This opening is, according to J. F. Meckel, (Manual of Anat. vol. 2 p. 220,) constantly found where the pulmonary artery gives off the descending aorta, and the vessel is said, in this case, to arise from both ventricles. It will be perceived that the effect of this malformation of the heart and anomalous distribution of its vessels, would have been, in a fœtus possessed of a perfect organization in other respects, after birth the distribution of pure arterial blood to the head and superior extremities, and of mixed arterial and venous to the inferior members and trunk.

The alimentary canal, the organs of secretion, of respiration, and the genito-urinary system were normal, as was also the remaining portion of the vascular system; if we except one defect, slight in character, but which to omit nothing, may be mentioned; which consisted in the passage of the ductus venosus from the umbilical to the left hepatic vein, instead of arising, as is most usual, from the vena porta.

## Figures and Tables

**Figure f1:**